# Biomechanical comparison of all-polyethylene total knee replacement and its metal-backed equivalent on periprosthetic tibia using the finite element method

**DOI:** 10.1186/s13018-024-04631-0

**Published:** 2024-02-23

**Authors:** Vasileios Apostolopoulos, Petr Boháč, Petr Marcián, Luboš Nachtnebl, Michal Mahdal, Lukáš Pazourek, Tomáš Tomáš

**Affiliations:** 1 First Department of Orthopaedic Surgery, St. Anne’s University Hospital and Faculty of Medicine, Masaryk University, Brno, Czech Republic; 2 Institute of Solid Mechanics, Mechatronics and Biomechanics, Faculty of Mechanical Engineering, University of Technology, Brno, Czech Republic

**Keywords:** Total knee arthroplasty, Computational modeling, Finite element method, All-polyethylene tibial component, Metal-backed tibial component, TKR, Knee replacement, FEA

## Abstract

**Background:**

Total knee arthroplasty (TKA) with all-polyethylene tibial (APT) components has shown comparable survivorship and clinical outcomes to that with metal-backed tibial (MBT). Although MBT is more frequently implanted, APT equivalents are considered a low-cost variant for elderly patients. A biomechanical analysis was assumed to be suitable to compare the response of the periprosthetic tibia after implantation of TKA NexGen APT and MBT equivalent.

**Methods:**

A standardised load model was used representing the highest load achieved during level walking. The geometry and material models were created using computed tomography data. In the analysis, a material model was created that represents a patient with osteopenia.

**Results:**

The equivalent strain distribution in the models of cancellous bone with an APT component showed values above 1000 με in the area below the medial tibial section, with MBT component were primarily localised in the stem tip area. For APT variants, the microstrain values in more than 80% of the volume were in the range from 300 to 1500 με, MBT only in less than 64% of the volume.

**Conclusion:**

The effect of APT implantation on the periprosthetic tibia was shown as equal or even superior to that of MBT despite maximum strain values occurring in different locations. On the basis of the strain distribution, the state of the bone tissue was analysed to determine whether bone tissue remodelling or remodelling would occur. Following clinical validation, outcomes could eventually modify the implant selection criteria and lead to more frequent implantation of APT components.

## Introduction

Total knee arthroplasty (TKA) is a surgical procedure that involves the anatomical replacement of the damaged knee with an artificial joint, considered an effective and the only definitive treatment of knee osteoarthritis. Total knee endoprostheses are most often divided in terms of the tibial component type, into all-polyethylene (ΑPT) and metal-backed (MBT). In modern knee arthroplasty, APT equivalents are considered a low-cost variant for elderly and low-demand activity patients [1]. APT implantations are estimated to be less than 1% of all TKAs according to several national joint replacement registries [2, 3].

**Fig. 1:**
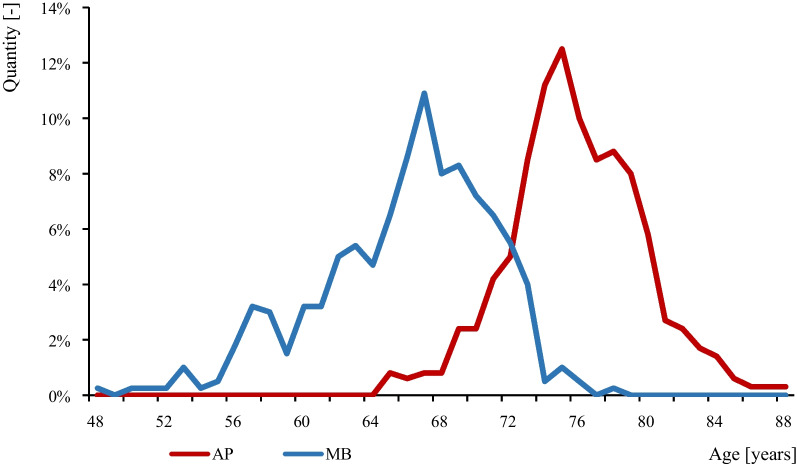
Patient age on the day of implantation of TKA NexGen APT and MBT at the First Department of Orthopaedic Surgery of St. Anne’s University Hospital

The clinical outcomes of TKA with APT have been described to be comparable to or even better than those with MBT [4, 5]. In our institution, the average age of the patients on the day of implantation of TKA NexGen APT was 75.4 years and only 12% were younger than 72 years. On the contrary, the average age of the patients on the day of implantation of TKA NexGen MBT was 65.9 years and only 11.75% were older than 72 years (Fig. 1) [4].

Previous biomechanical analysis demonstrated, using the finite element method, that APT in patients of the 60–70-year age group showed a similar induced mechanical response. Moreover, APT was shown to induce remodelling and modelling of the periprosthetic tibia. As a result, more frequent implantation of APT in younger patients was suggested [6].

There are no evidence-based biomechanical guidelines for orthopaedic surgeons to consider when choosing the tibial component, especially in terms of patients’ age and bone quality. The purpose of this study is to biomechanically evaluate and compare the response of the periprosthetic tibia after the implantation of TKA NexGen APT CR and TKA NexGen MBT CR.

FEM simulations can be used to examine the stresses and strains experienced by the bone tissue around the TKA implant and to investigate the potential effects of implant design changes or surgical techniques on the surrounding bone tissue [7, 8]. Investigation of the mechanical response of the periprosthetic tibia by means of an in vivo examination is not feasible. For this reason, an in silico approach was chosen. Tibia models of three defined age categories were created as well as a tibia model corresponding mechanically to osteopenia. The assumption that APT can offer a similar or even better mechanical response of the periprosthetic tibia of 60–70-year age groups, could lead to more frequent implantation of TKA APT in younger patients and lowering of the indicative age limit.

## Materials and methods

Computational modelling allows the simulation and analysis of states, which would be difficult to achieve experimentally (due to non-physiological loading) and helps optimise the design behaviour of components. It is also a very useful tool for the prediction of conditions in orthopaedics and demonstrates an effective preoperative method for planning patient-specific TKA implantation [9, 10].

In this work, bone tissue models of the geometry of the tibia were created from a set of computed tomography (CT) images of a representative patient, who was carefully selected as an optimal example due to his diagnosis, age, and bone tissue status. This representative male patient was 65 years old at the time the CT images were obtained. The patient was diagnosed with 3rd-grade osteoarthritis of the knee joint according to Kellgren–Lawrence classification; otherwise, the patient did not suffer from any other illness that would affect the state of bone tissues or their geometry [11]. Models of the geometry of technical components were created based on real components through a reverse engineering approach.

The complete knee endoprosthesis and bone tissue were both represented in the computational model. The material characteristics and patient-specific tibia bone geometry were taken into consideration in computational simulations of the mechanical performance of TKA. To compare APT and MBT, we used one of the most widely used and clinically proven total knee systems in the world [12]. The NexGen CR prostheses have a similar geometric design in both the MBT and the APT, and have the same corresponding femoral component [9]. The following subsections provide further details about computational modelling.

### Model of geometry

To obtain image datasets of the tibia of a representative patient, a CT scanner (LightSpeed VTC, General Electric, Boston, MA, USA) with a voxel size of 0.7031 mm × 0.7031 mm × 0.625 mm was used. The CT images were manually segmented in the application programmed in the MATLAB 2012 environment (Math Works, Natick, MA, USA) [13]. Using this procedure, Standard Tessellation Language (STL) files were created. Using a 3D scanner (Shining3D EinScan SE, SHINING 3D Technology GmbH, Stuttgart, Germany), STL format files of all TKA components were obtained.

**Fig. 2:**
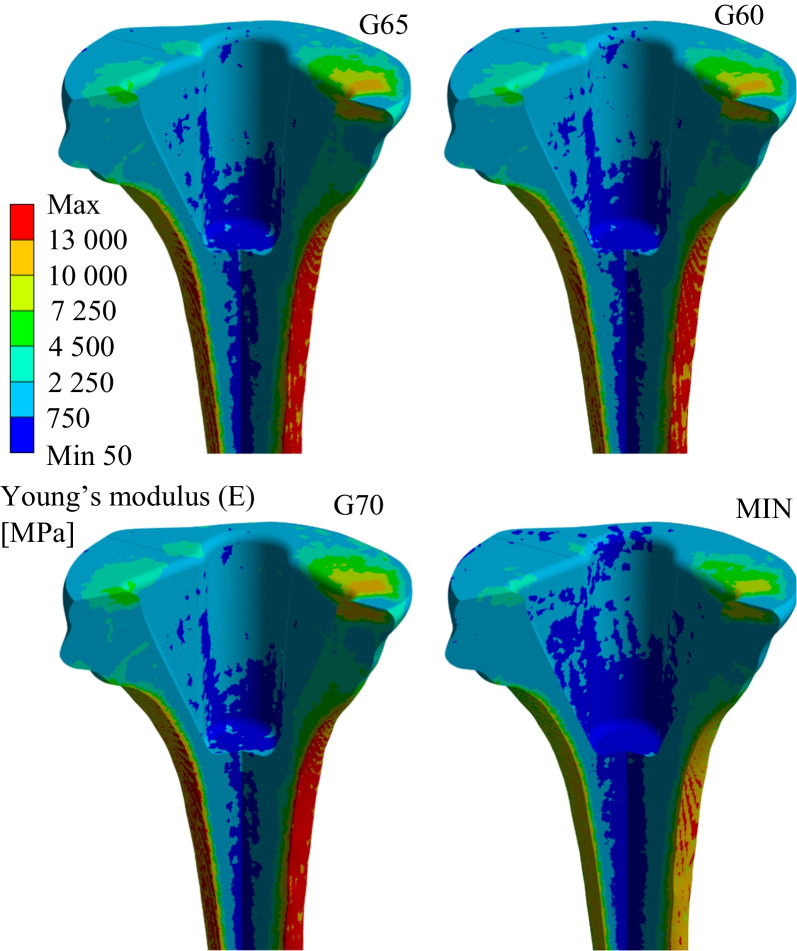
Young’s modulus distribution in the cancellous bone tissue model for each analysed group

All STL files were further processed in SpaceClaim ANSYS® Academic Research Mechanical, Release 22.2 (Swanson Analysis, Inc., Houston, PA, USA). The resulting geometry of the assembly is shown in Fig. 2.

**Fig. 3:**
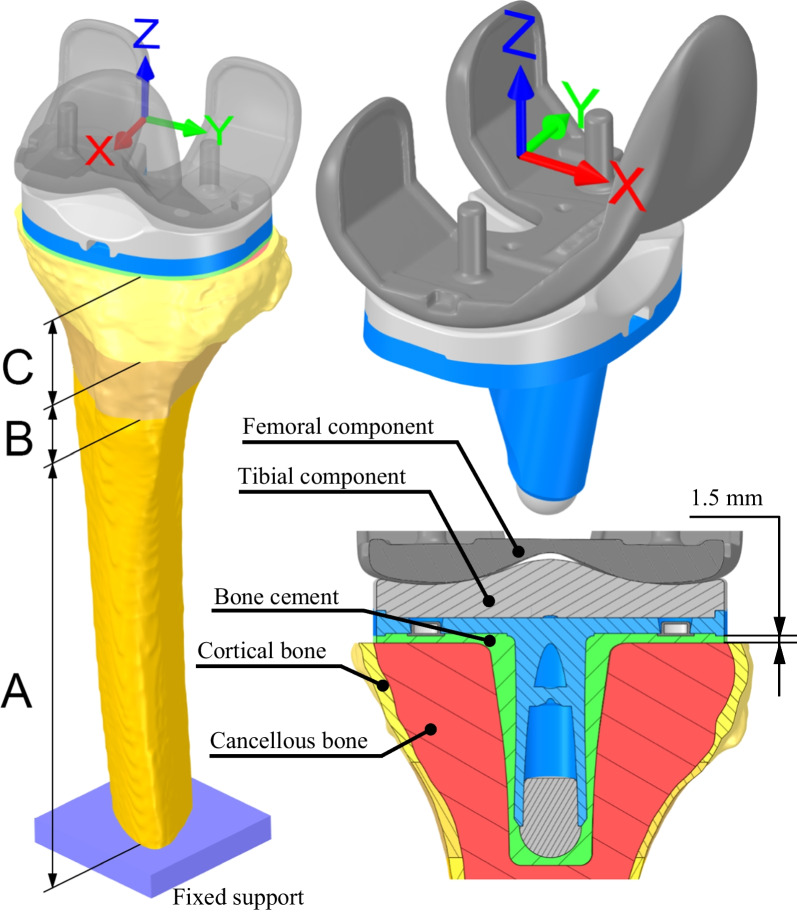
Model of geometry with boundary conditions shown on the MB TKA variant

In total, one tibia model, both types of tibial components, one common femoral component, and two volume models of bone cement corresponding to the given type of tibial component were created. The principle of mechanical alignment has been used for the assessment of the tibia cut. The size of the tibial and femoral components were chosen on the basis of the manufacturer's catalogue data and instructions and to match the dimensions of the modelled knee-joint. Specifically, the tibial size of 6 was chosen for the polyethylene monoblock with a height of 10 mm and an equivalent size of 6 for the tibial metal tray with 10 mm-high polyethylene inlay. In case of the femoral component, size “G” was chosen in correspondence with the tibial component [9]. The hole in the tibia and position of the tibial component were set at 4° of external rotation compared with the tubercle landmark and 7° of posterior slope based on the procedures used during surgery recommended by the manufacturer [9, 14]. The femoral component flexion has been set 4° to the tibial component [15, 16]. Based on TKA cementing technique, the design of geometry models of the bone cement was set so that their outer boundaries remained within the outer boundaries of the cortical bone tissue model and aligned with the outer edge of the tibial component's geometry. Furthermore, it was considered that the bone cement would fill the created opening in the cancellous bone tissue and be in full contact with the tibial component [17]. The dimensions that will be in contact with the bone tissue volume models of the analysed APT and MBT variants of bone cement volume models are the same. The distance from the edge of the tibial component to the surface of the tibial cut was modelled with a dimension of 1.5 mm (see Fig. 3).

### Meshing procedure

All solid models were discretised in ANSYS® by using quadratic hexahedral and quadratic tetrahedral elements (element types SOLID186 and SOLID187). Contact surfaces were meshed by using contact elements CONTA174 and TARGE170. The mesh consisted of approximately 3.5 million elements in both cases. The global size of the elements for cancellous and cortical bone tissue was 1 mm, for the tibial component it was 2 mm, for bone cement it was 0.5 mm, and for the femoral component it was 2 mm. The sizes of the elements were selected based on the preliminary tests and sensitivity calculations [6].

### Material model

All materials were considered linearly elastic and isotropic. The cancellous bone tissue material model was considered heterogeneous. All other material models were considered homogeneous. CT images were used to determine material models of both cancellous and cortical bone tissues. In total, 60 sets of CT images from different patients were assessed and statistically processed: 45 in previous study [6] and an additional 15 in this study. The result from the statistical evaluation of HU values in the cancellous bone tissues was the dependency of the global change of Young's modulus values of the cancellous bone tissue material model. These 60 sets of CT images were divided into four groups according to age, each group containing 15 patients. The first group of patients aged 59–61 years was referred to as ‘G60’, the second group aged 64–67 years was referred to as ‘G65’, and the third group aged from 70 to 72 years was referred to as ‘G70’. The last group, referred to as ‘MIN’, represents patients aged from 65 to 72 years with diagnosed osteopenia (lower bone density). For the ‘MIN’ group, we have evaluated 15 CT datasets following the same approach as in previous study [6], for calculations we have used formulas 1,2,3.

Apparent density [18]

1 $$\rho =114+0.916\cdot \left(\frac{{\text{kg}}}{{{\text{m}}}^{3}}\right)$$

Cortical bone Young’s modulus [18]

2 $$E=-3.842+0.013\cdot \rho \left({\text{GPa}}\right)$$

Cancellous bone Young’s modulus [18]

3 $$E=\frac{0.51\cdot {\rho }^{1.37}}{1000} \left({\text{GPa}}\right)$$

In total, four cancellous bone tissue material models were created (Fig. 2). Based on a previous study, the material model of cortical bone tissue was divided into three parts labelled ‘A’, ‘B’, and ‘C’, each assigned with its own Young’s modulus [6]. Table 1 shows the values of the prescribed material characteristics (Table 2).

**Table 1:** Material properties for each part of the assembly

Part	Material	E [MPa]	µ [−]	References
All-poly components	UHMWPE	670	0.46	[19]
Metal part of the MBT component	Tivanium® alloy	110,000	0.30	[9, 20]
Femoral component	Zimaloy®	210,000	0.29	[9, 21]
Bone cement	Palacos R	2891	0.40	[22]
‘A’—155 mm	Cortical bone	15,000	0.30	[6, 18]
‘B’—20 mm	Cortical bone	10,500	0.30	[6, 18]
‘C’—25 mm	Cortical bone	7500	0.30	[6, 18]
Cancellous bone	Cancellous bone	Heterogeneous	0.30	[6, 18]

**Table 2:** Applied forces and rotations

Axis	Force [N]	Rotation [°]
X	0.0291·BW	1.08
Y	− 0.1623·BW	3.08
Z	− 2.6807·BW	3.16

A heterogeneous distribution of Young’s modulus was generated from a set of CT images of a representative patient with the same procedure described in the previous study [6]. The mapping was performed using the in-house software CTPixelMapper programmed in Python 3.4 [23].

Since the representative patient belongs to the ‘G65’ group, this group is considered a reference for global changes in Young’s modulus values of the entire cancellous bone tissue material model. Based on the observations made in this and the previous study [6], Young’s modulus values of the material model for the ‘G60’ group were globally adjusted by + 5% compared to the ‘G65’ reference group. The ‘G70’ group had a globally reduced value of Young’s modulus of − 5% compared to the ‘G65’ reference group. The last group, ‘MIN’, with diagnosed osteopenia had a value of Young’s modulus globally reduced by 21% compared to group ‘G65’. A similar process of Young’s modulus reduction has been used for the osteoporotic bone tissue material model [24]. Figure 2 shows all created variants of cancellous bone tissue material models.

### Loads and boundary conditions

The load which is considered standardised was applied to all internal surfaces of the femoral component that come into contact with bone cement or bone tissue after implantation [25]. Based on the data provided by the authors, the greatest load on the knee joint occurs in 39% of the level walking phase. The loading condition was applied trough a pilot point (PN) with precisely determined position, based in the centre of the coordinate system, described in the publication [25]. The PN was connected with multipoint constraint (MPC) with all internal surfaces mentioned above. This condition was modelled using MPC capabilities of the contact elements. The position of the femoral component was set following the recommended surgical technique of the manufacturer to distribute the contact surfaces as symmetrically as possible across the polyethylene liner [9]. The contact pairs between the femoral components and the tibial components of the TKA were modeled as frictional contact with a coefficient of friction (f) of 0.05 (−) [26]. The contact pairs between the cement and the tibial components were modelled as bonded contact as was contact between the cement and bone tissues.

Table 3 lists the applied load values [25]; these values are relative to the coordinate system shown in Fig. 3. Values are presented as multiples of body weight (BW) by a factor of g (g = 9.81 ms^−2^). The weight of the representative patient was 80 kg [6].

**Table 3:** Simulations overview

Tibial Component	Designation based on groups	Material model		Model of geometry, mesh	Load and boundary conditions
		Cancellous bone tissue	Cortical bone tissue		
AP	G60	E + 5% from G65	Same for all models	Common to all groups and AP TKA	Same for all models
	G65	Based on representative patient			
	G70	E − 5% from G65			
	MIN	E − 21% from G65			
MB	G60	E + 5% from G65		Common to all groups and MB TKA	
	G65	Based on representative patient			
	G70	E − 5% from G65			
	MIN	E − 21% from G65			

### Simulations overview

In this study, we have analysed two different types of TKA, and for each type, we have created four variants, which are shown in Table 3.

These models were used for evaluations of equivalent (von Mises) strain in the bone. These evaluations follow the common practice used in musculoskeletal computational biomechanics and they are based on the Mechanostat hypothesis [27–29]. This hypothesis works with Wolf's law, which says that strains induced in the bone tissues affect the bone architecture. According to the Mechanostat hypothesis, certain ranges of strain values can positively or negatively affect bone modelling and remodelling. Since the main focus is on the strains in tibia, stresses in TKA are not analysed in this paper.

## Results

### Equivalent strain

**Fig. 4:**
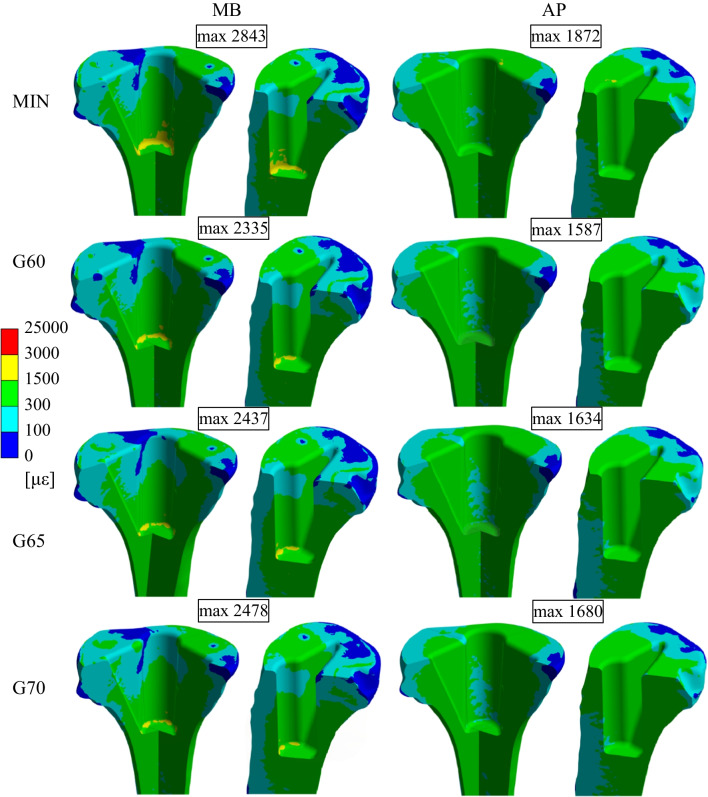
Equivalent strain distribution for all analysed groups in frontal and sagittal view

Figure 4 shows the equivalent microstrain distribution in two different views (frontal and sagittal). The microstrain (με) is defined as 1000 με $=$ 0.1% change in length. All the results of the equivalent microstrain distribution are presented in specific intervals based on the Mechanostat [27–30]. It is apparent that the greatest strain values occur under the tibial cut on the medial side of the bone in the case of the APT component. On the other side, in the case of the MBT component, the greatest strain values occur under the stem tip. The maximum microstrain values for all groups are below the critical 25,000 με [30], with the highest values of 2843 με using the MBT component and 1872 με using APT component, both occurring in the MIN group.

**Fig. 5:**
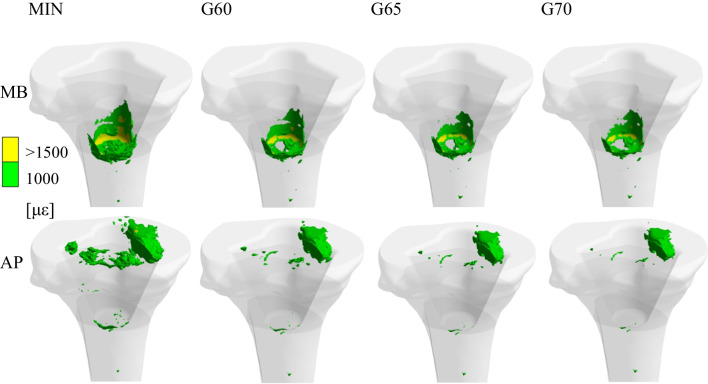
Values of equivalent strain over 1000 με shown in the volume of each analysed variant

Figure 5 shows the equivalent microstrain distribution in the volume model of the cancellous bone tissue for each analysed variant. It is visible that in the case of the model with the APT component, the microstrain values above 1000 με are in the area below the tibial section and are located rather in the medial area of the tibia. In the case of the model with an MBT component, microstrain values above 1500 με are mostly localised in the stem tip area. Even in this case, the microstrain values are higher on the medial side; however, this phenomenon is not as significant as in the model with the APT component.

**Fig. 6:**
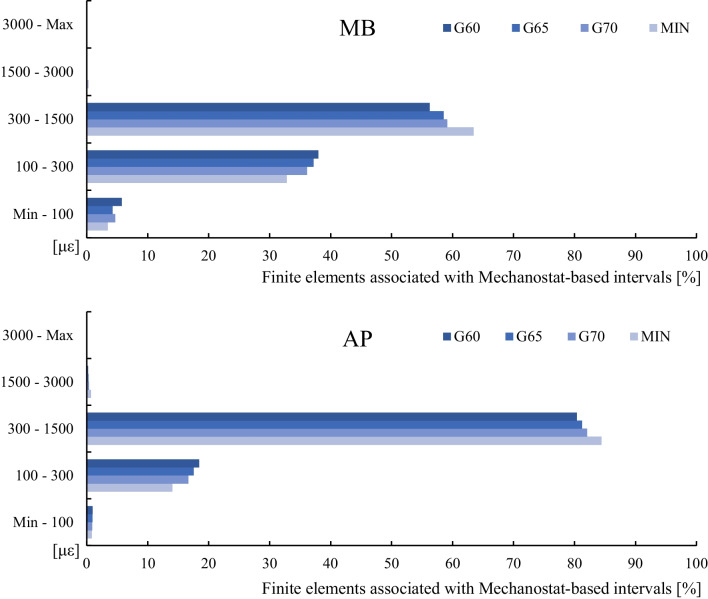
Percentage of periprosthetic bone volume that exhibited strains associated with a specific interval of the Mechanostat

Figure 6 shows a graph with the values of the microstrain for each tibial component and each variant of the cancellous bone tissue material model. These values were analysed in a volume section determined by a distance of 55 mm from the tibial cut.

It is visible that for the variants with the APT component and previously described three age groups (G60, G65, G70), the microstrain values in more than 80% of the volume are in the range from 300 to 1500 με; for the group MIN the same range of microstrain values can be found in more than 84% of the volume; and the maximum microstrain values in the range of 1500 to 3000 με occur in less than 0.75% of the volume for all cases.

For variants with the MBT component and the three age groups, the microstrain values are mainly in the range from 300 to 1500 με in more than 56% of volume and in the range from 100 to 300 με in more than 36% of volume. For the group MIN, microstrain values in the same ranges can be found in more than 63% and 32% of the volume, respectively. In the case of the model with the MBT component, the microstrain values are in the range from a minimum to 100 με in more than 3.4% of the volume, while in the case of the model with the APT component, this range of values occurs only in less than 1% of the volume.

## Discussion

Clinical studies have demonstrated that there is no significant difference between APT and MBT performance in terms of survivorship, clinical outcomes, range of knee motion, and rate of revision [31, 32]. MBT is preferred, and more frequently implanted [2, 3]. This could be explained by the greater intra-operative flexibility of MBT, and the ability to revise the replacement with an exchange of the polyethylene insert, without having to extract the tibial component [33]. Although, due to the growing number of replacements, and the significantly lower cost of APT components, there has been renewed interest in using them considering the economic strain on healthcare [34].

In the past, several studies have compared the AP and MB tibial components and their effect on tibial bone tissue [35, 36]. In this study, the load was applied through a designated femoral component, which is not commonly used. Since the load was applied through this component, it was possible to use the so-called standardised up-to-date knee joint load model [25]. The study used an ultracongruent implant with anterior increased congruence of the same manufacturer (Innex, Zimmer) that can be used also as cruciate sacrificing, and the same femoral component as the CR equivalent [25, 37]. This increased anterior congruence could provide additional constrain against the anterior sliding when posterior cruciate deficiency is present, but not in the examined phase of gait [37, 38]. As a result, the applied loading from the femoral component could be also used for CR equivalents. The load model used does not give a specific percentage distribution of the force acting on the medial and lateral sides, as is usual. Rotations of the femoral component were also included in this loading model. The resulting force distribution in this study was approximately 40% on the lateral side and 60% on the medial side of the tibia, which is consistent with commonly used loading models [39, 40]. To minimalise the bias, we have not included the patellar ligament from the model of the load due to its insignificant acting force below 50 N during the specified gait cycle moment [41, 42]. According to the literature, the posterior cruciate ligament can act with a force of 0–20 N, which has been also considered insignificant, and not included in the presented load model [38, 43]. Also, the lateral and medial collateral ligament has been not included to our load model due to their minimal induced forces between 10 and 50 N during the analysed part of the gait cycle [38, 42, 44]. Based on the results from the authors of the standardized load model, all acting loads in TKA during gait cycle are applied through the PN in the centre of the coordinate system.

In this study, geometry and material models were used that correspond to typical patients who should undergo TKA. All material models of biological tissues were carefully determined based on statistical analysis of CT image measurement sets of selected representative patients in given age groups. This study, in conjunction with previous analysis [6], tries to provide a large amount of objective information about the methodology that can be reproduced and used to analyse other types of TKA.

To ensure that the examination applies to a wide range of patients, we created a cancellous bone tissue material model representing patients with diagnosed osteopenia. In total, we evaluated 15 sets of CT images of osteopenic patients, with diagnosis confirmed previously with dual-energy X-ray absorptiometry [45]. Depending on measured Hounsfield units, we reduced the Young’s modulus value of cancellous bone by 21% compared to that in group ‘G65’. There is a high prevalence of osteopenia in elderly patients undergoing TKA and it presents an increased risk of perioperative complications [46]. Osteoporosis was not preferred due to changes in the bone microarchitecture, wide density variation, and the absence of a lower bone density limit; on the contrary, osteopenia is strictly defined [47]. In cases of severe osteoporosis, the indication of TKA implantation is questionable because of fracture risk and decreased osteointegration ability [48].

The results for the variant with an APT component show the highest equivalent strain values in the area of the tibial cut and its immediate vicinity. In the majority of the volume of periprosthetic bone tissue, the equivalent strain value is in the range 300–1500 με. This determined range corresponds to physiological loading and moderate overloading that induces the modelling and remodelling of bone tissue. None of the solved variants with the APT component show a higher value of equivalent strain than 1872 με in case of MIN group, presenting a strain value that induces modelling and remodelling of bone tissue [28, 29]. This could contribute to a subsequent reduction in the risk of aseptic loosening. The results for the variant with an MBT component show the highest equivalent strain values around the bottom of the hole created for the tibial component in the tip of the tibial component stem. In the majority of the volume of periprosthetic bone tissue, the equivalent strain is generally lower than with the APT component; however, the range of values is similar. In all analysed variants with the MBT component, there was a region of higher equivalent strain values, namely in the area under the tip of the tibial component stem. In this area there could be an increased risk of pathological overloading, especially during sport and physical activities that may lead to aseptic loosening of tibial component [49]. In the majority of the volume of periprosthetic bone tissue, the calculated equivalent strain was supposed to support the modelling and remodelling of bone tissue. However, the equivalent strain volume percentage inducing bone modelling and remodelling is generally lower than when compared with the APT component. In accordance with our study, older studies using finite element modelling also showed that strain distribution on the cancellous bone may be lowered if an MBT component is employed [35, 50].

The strengths of this study include the use of boundary conditions which are considered standardised, the use of implants from a single manufacturer (Zimmer) comparing a modern congruent APT component to a modular MBT component of the same design, the same implantation technique, and the bone tissue model. Those attributes allowed an assessment and comparison of the implants excluding possible variables that could negatively affect the validity of the results. On the contrary, the knee geometry model is patient-specific: the created geometry is based on a CT dataset of a 65-year-old patient with advanced knee arthritis. The contact between the bone cement and the tibia is assumed to be completely bonded. Also, the defined boundary conditions simulate only a single possible load (maximum) appearing in the gait cycle. Finally, the results of this finite element analysis require further mechanical experiments or clinical validation.

## Conclusion

In summary, this study compared the response of the periprosthetic tibia after the implantation of TKA NexGen APT and its MBT equivalent, by using standardised implant loading. Considering limitations, we appraise the effect of APT implantation on the periprosthetic tibia to be equal or even superior to that of MBT despite maximum strain values occurring in different locations. On the basis of the strain distribution, the state of the bone tissue was analysed to determine whether bone tissue remodelling or remodelling would occur. With respect to our computational model, we purpose that APT components would have induced beneficial strain distribution of bone tissue in a greater volume of periprosthetic tibia than MBT for all defined groups. Following clinical validation, outcomes could eventually modify the implant selection criteria and lead to more frequent implantation of TKA with APT components.

## Data Availability

The data that support the findings of this study are available from the corresponding author upon reasonable request.
